# Prevalence and determinants of non-adherence to Imatinib in the first 3-months treatment among newly diagnosed Ethiopian’s with chronic myeloid leukemia

**DOI:** 10.1371/journal.pone.0213557

**Published:** 2019-03-07

**Authors:** Atalay Mulu Fentie, Fishatsion Tadesse, Ephrem Engidawork, Am Gebremedhin

**Affiliations:** 1 School of Pharmacy, College of Health Sciences, Addis Ababa University, Addis Ababa, Ethiopia; 2 School of Medicine, College of Health Sciences, Addis Ababa University, Addis Ababa, Ethiopia; European Institute of Oncology, ITALY

## Abstract

**Objectives:**

Imatinib has shown to be highly efficacious in the treatment of chronic myeloid leukemia (CML) but continuous dosing and patient adherence is essential treatment success. The study aimed to assess prevalence and reasons for non-adherence to Imatinib in newly diagnosed patients with CML in the first 3-months of treatment.

**Methods:**

The study was conducted from October 1, 2016 to November 30, 2017 at Tikur Anbessa Specialized Hospital, Addis Ababa, Ethiopia. A total of 147 newly diagnosed patients were followed and their adherence status was determined using the 8-items Morisky Medication Adherence Scale and reasons for their non-adherence were evaluated using semi-structured questionnaire. Descriptive statistics were used to summarize the data while multivariable logistic regression was employed to explore associations among variables of interest.

**Results:**

Participants’ median age at time of confirmed diagnosis was 36 years; with most of them in the age group of <40 years (64.6%). Males comprised 59.2%. Adherence rate was found to be 55.1%. Those who lived in rural area, had low income, adverse drug events and comorbidity were significantly associated with treatment non-adherence. Most (68.4%) patients missed their medication due to adverse drug events. Three patients were lost-to-follow-up. Among 144 patients who finished the 3-month follow-up, 91.7% of them achieved complete hematologic remission. Morisky high adherent (AOR = 8.6, 95%CI:4.32–11.1) was positively associated with complete hematologic remission.

**Conclusions:**

Overall treatment adherence is suboptimal. Thus, efforts should be made to improve adherence and further study is required to explore impact adherence on the cytogenetic and molecular responses of Ethiopian patients with CML.

## Introduction

The history of CML treatment has undergone different paradigm shifts from Arsenic trioxide in the 19^th^ century to radiotherapy, allogenic stem cell transplantation, recombinant interferon-alfa, Busulphan and Hydroxyurea; and now more recently and importantly to with tyrosine kinase inhibitors(TKIs) [1,2].

Imatinib mesylate (Imatinib) was the first TKI to receive approval by the Food and Drug Administration (FDA) for the treatment of patients with Philadelphia chromosome positive (Ph+) CML in 2001 [3]. This was a major advance in the pharmacologic treatment of CML with regard to efficacy and safety with improved outcomes [4]. It acts via competitive inhibition at the ATP-binding site of the BCR-ABL tyrosine kinase, which then results in inhibition of phosphorylation of downstream proteins involved in cell signal transduction [5,6].

Although Imatinib improves overall survival rate, continuous and adequate dosing is required for achieving optimal response. This, besides improving the overall response of the patient, it also limits or avoids altogether additional health care costs associated with the management of disease progression [7]. Hence, patient adherence, the extent to which patients take their medications as prescribed by their health-care provider, with respect to timing, dosage, and frequency [8], is critical for better treatment outcomes. Although strict adherence to treatment is known to be crucial, many studies have demonstrated that poor adherence to Imatinib therapy is frequent, and thus may significantly affect therapeutic outcomes [9,10].

Therefore, frequent assessment of adherence and early identification of contributing factors to poor adherence can help in plan interventions to overcome the barriers and, therefore, improve treatment response and outcomes. Hence, this study was undertaken to obtain information on adherence to treatment of CML patients taking Imatinib and to document factors that are involved in poor adherence and then suggest mechanisms that may mitigate poor adherence to treatment in the long-term.

## Patients and methods

The study was conducted at the outpatient hematology clinics of Tikur Anbessa Specialized Hospital (TASH), a tertiary care center affiliated with College of Health Sciences, Addis Ababa University. All patients with CML from all regions of the country are referred to this hospital to be enrolled in the Glivec International Patient Assistance Program (GIPAP). Under this program, patients get free access to treatment with Imatinib and second generation TKIs and are followed closely and regularly in the outpatient clinics of the division of Hematology in the Department of Internal Medicine. On average, 3–5 new CML cases were diagnosed per week during the study period.

Ethical clearance was taken from the Ethical Review Board of School of Pharmacy and approval of the study protocol was obtained in the hematology division of Internal medicine department. Prior to data collection, individuals were informed about the study and verbal assent was taken from parents for those study participants whose age was <18 years and verbal consent was obtained from the rest of the study participants. But as rule and part of GIPAP, every patient taking Imatinib must sign the written informed consent. Written and verbal consent or assent procedures were first approved by ethics review board of our institution. Each patient was informed about the objective of the study, procedures of selection and assurance of confidentiality. Individuals were informed that it is fully voluntary and they can withdraw from the study at any time and this would not affect the service they get from the hospital. They were also informed that they would not receive any monetary incentive for participating in the study. The collected data was secured in a lockable cabinet, no identifiers were used and data was analyzed in aggregate to maintain confidentiality and anonymity of information.

A prospective cohort study was employed and was started in October, 2016. Based on the inclusion criteria a total of 147 newly diagnosed patients with CML and who started Imatinib treatment between October 1, 2016 to September 1, 2017 were included in the analysis and their adherence status and a preliminary 3-month treatment response statuses were presented. Each patient was followed for 3- month and the outcome variable was determined at the end of follow-up.

A pre-tested, semi-structured questionnaire and 8-item Modified Morisky Adherence Scale(MMAS-8) [11] were used to extract information of interest. The data abstraction form was also used to capture baseline and follow-up information from patient chart. It had two parts. Part I was for all baseline findings at time of diagnosis and at initiation of Imatinib treatment. Part II was for follow-up findings. Information was categorized to those findings obtained at time of confirmed diagnosis, at initiation of Imatinib treatment and follow-up findings. The semi-structured questionnaire was used to ask their willingness to participate in the study and; gather information on socio-demographic characteristics, reasons for non-adherence to Iamtinib and encountered adverse drug events. The MMAS-8 is a 7 items with yes/no response options and 1 item with a 5-point likert scale response option. MMAS-8 is a part of the WHO-case management adherence guideline assessment tools and mostly used to classify patients as “low-patients who scored <6”, “medium- score <8 and ≥6” and “high-score 8” on motivation and knowledge domain. Patient reported reasons to medication non-adherence was collected for those patients whose adherence status was sub-optimal(i.e either in medium or low adherent category). Documented objective reasons for non-adherence and adverse effects encountered were also collected from patient chart. In addition treatment history was also collected by using data abstraction format designed as per the appointment period’s given for each particular patient.

Data were analyzed by using SPSS version-21. Descriptive statistics were used to summarize socio-demographic data, clinical and treatment related characteristics. Bivariate logistic regression analysis was conducted to see the existence of association of adherence to Imatinib treatment with independent variables. All variables with *p*-value<0.25 in univariate analysis were included in the multivariable binary logistic regression, and *p*-value < 0.05 was considered as statistically significant. The χ2 and fisher exact tests with significance level of p< 0.05 were employed to explore differences in proportions of complete hematologic response achievement between length of treatment and white blood cell count category at initiation of Imatinib.

## Results

A total of 147 eligible newly diagnosed patients with CML were enrolled during the study period. Their mean age was 37.8 years (SD = 13.7) and median 36 years (Range 14–74); with most (65.3%) of the patients being in the age group of less than 40 years. Majority of the patients were male (59.2%), with male to female ratio of 1.45:1 and married (68.7%). Being employee (25.2%), attended secondary education (29.3%) and do not read and write (29.3%) accounted the highest proportion. Most patients (74.8%) were out of Addis Ababa. Almost half (45.6%) of the study participants were > 300km away from the Hospital. A significant proportion of the study participants (48.3%) were in a very low or low level of monthly family income (Table 1).

**Table 1:** Socio-demographic characteristic of newly diagnosed patients with CML in Ethiopia from October 1, 2016- September 1, 2017.

Variables		Phase of CML, N (%)		
		CP (n = 136)	AP(n = 11)	Total (n = 147)
**Age (in years)**				
		Median(Range), 36(14–74)			
		Mean ± SD, 37.8 ± 13.7			
**Sex**				
	Male	81(59.6)	6(54.5)	87 (59.2)
	Female	55(40.4)	5(45.5)	60 (40.8)
**Marital Status**				
	Single(single, divorced and widowed)	42(30.9)	4(36.4)	46(31.3)
	Married	94(69.1)	7(63.6)	101(68.7)
**Educational Status**				
	Can’t read and write	41(30.1)	2(18.2)	43(29.3)
	Primary Education	33(24.3)	4(36.4)	37(25.2)
	Secondary Education	21(15.4)	1(9.0)	22(14.9)
	Higher Education	39(28.7)	4(36.4)	43(29.3)
	Can read and write but have no formal education	2(1.5)	0(0)	2(1.3)
**Occupation**				
	Farmer	24(17.6)	1(9.1)	25(17.0)
	Merchant/Trader	23(16.9)	3(27.3)	26(17.7)
	Employed^a^	33(24.3)	4(36.4)	37(25.2)
	House wife	21(15.4)	2(18.1)	23(15.6)
	Retired	15(11.1)	0(0.0)	15(10.2)
	Students	20(14.7)	1(9.1)	21(14.3)
**Residence, Region**				
	Addis Ababa	36(26.5)	1(9.1)	37(25.2)
	Oromia	35(25.7)	2(18.1)	37(25.2)
	Amra	33(24.3)	2(18.1)	35(23.8)
	SNNPR	13(9.6)	3(27.5)	16(10.9)
	Tigray	11(8.0)	1(9.1)	12(8.1)
	Others**	8(5.9)	2(18.1)	10(6.8)
**Average distance between Patients home & TASH**				
	< 100km	42(30.9)	1(9.1)	43(29.2)
	100-300km	33(24.3)	4(36.4)	37(25.2)
	>300 km	61(44.8)	6(54.5)	67(45.6)
**Monthly Family Income in ETB**^$^				
	Low and Very Low (≤1500)	68(50.0)	3(27.3)	71(48.3)
	Average and Above Average (1501–5000)	53(39.0)	7(63.6)	60(40.8)
	High (≥ 5001)	15(11.0)	1(9.1)	16(10.9)

^a^ Having a job working for a company or another person for a monthly salary basis

**Afar, Harari, Ethiopian Somali, Diredawa, Benishangul

^$^Based on the Ethiopian Civil Service monthly salary scale for civil servants.

**Fig 1:**
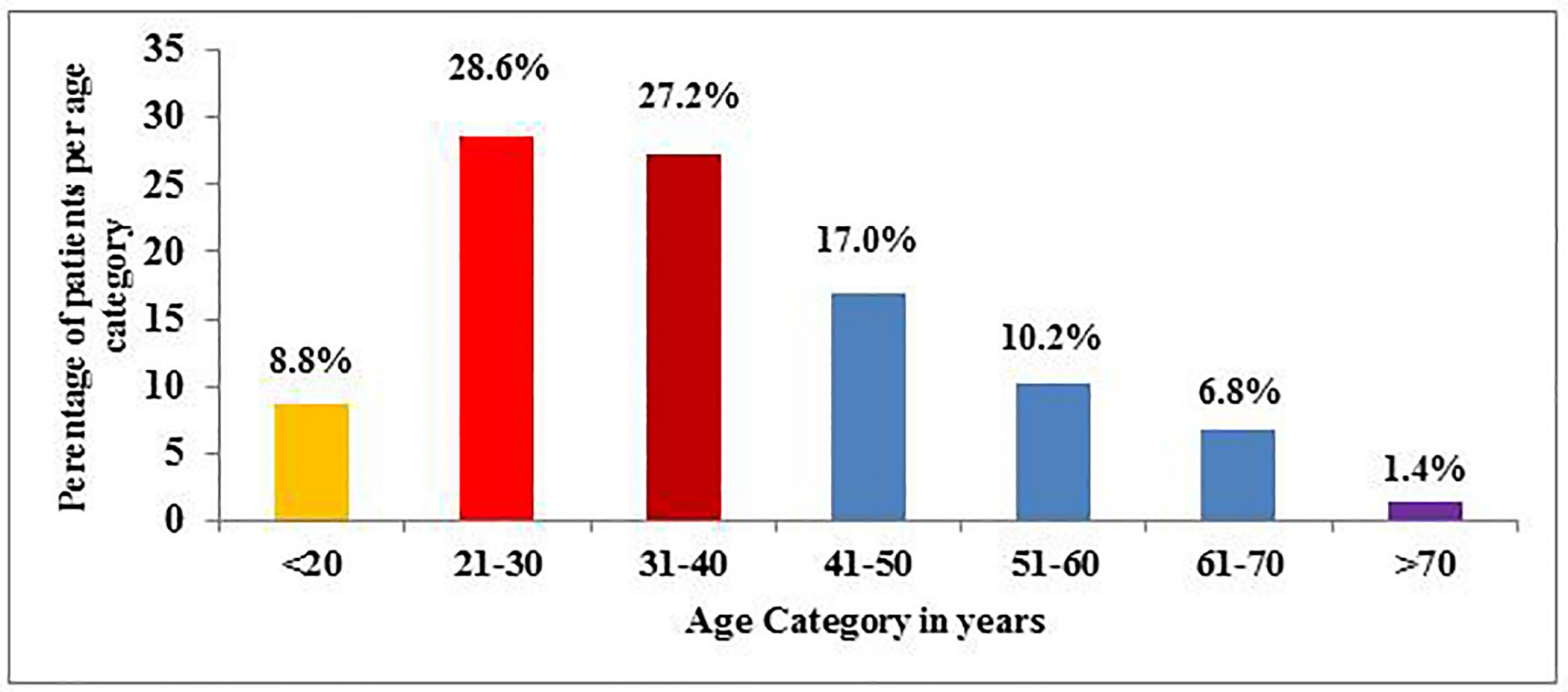
Age category in years.

Regarding age distribution of newly diagnosed patients with CML, majority 28.6% of them were in the 21–30 years age category; followed by 31–40 years 27.2% and 41–50 years, 17.0% (Fig 1).

Majority (92.5%) of the patients had taken upfront hydroxyurea treatment for cytoreduction. Treatment duration of hydroxyurea ranged from 2-weeks to 9.5 months, with median treatment duration of 1-month. In addition, the latency for initiation of Imatinib treatment between suspected diagnosis of CML through bone marrow and confirmed diagnosis by cytogenetic test ranged from 2-weeks to 1-year with median period of 1.5 months. Nearly, all patients (95.9%) received 400 mg Imatinib as a daily dose and 24.5% of them also had taken HU and Imatinib combination for a maximum of 2-months and a minimum of 2-weeks, with median of 2-weeks duration (Table 2).

**Table 2:** Treatment history at initiation of Imatinib treatment for newly diagnosed patients with CML in Ethiopia, from October 1, 2016- November 30, 2017.

Base line Findings at Initiation of Imatinib			Median (Ranges)	N	%
Treatment History					
	**For Cytoreduction treatment**				
		HU*		136	92.5
		No (directly started IM^a^ treatment)		11	7.5
		Duration of HU treatment, In months	1.0 (0–9.5)		
		Latency of Initiation of IM therapy	1.5(0.5–12.0)		
	**Initial daily Dose (IM)**				
		400 mg		141	95.9
		600 mg		6	4.1
	**IM + HU combination treatment**				
		Yes		36	24.5
		No		111	76.5
		Duration of combination, in months	0.5(0.5–2)		

*Hydroxyurea

^a^ Imatinib

Among the 147 study participants, management was altered in 35 of the patients. Alterations included were temporarily discontinuing treatment by physicians (n = 25) or due to stock out of Imatinib (n = 2), as well as decreasing dose of Imatinib due to intolerance (n = 5) or increasing dose as a result of poor response (n = 3). The median total duration of treatment discontinuation was 14 days (Range: 14–42 days). Most of the physician-led temporary treatment discontinuations were encountered within one month of therapy at median Imatinib treatment duration of 21 days (14–90 days). Almost all patients’ (n = 23) whose treatment temporarily discontinued had resumed treatment, except for two patients due to pregnancy at 2 and 2½ months of Imatinib treatment. Among the 30 study participants who fulfilled temporary treatment discontinuation or dose decrement due to safety issue, grade III/IV Imatinib toxicity accounted for the highest proportion (28, 93.3%). Grade III/IV thrombocytopenia was found to be the main reason (12, 40.0%) followed by neutropenia (7, 23.3%), bicytopenia (5, 16.7%) and skin rash (3, 10.0%). In addition, in two study participants, Imatinib was also temporarily discontinued due to grade III/IV thrombocytopenia though they didn’t fulfill the criteria of discontinuation. The rest two and one reasons were associated with being pregnant while on Imatinb treatment and severe arthralgia/myalgia, respectively.

**Fig 2:**
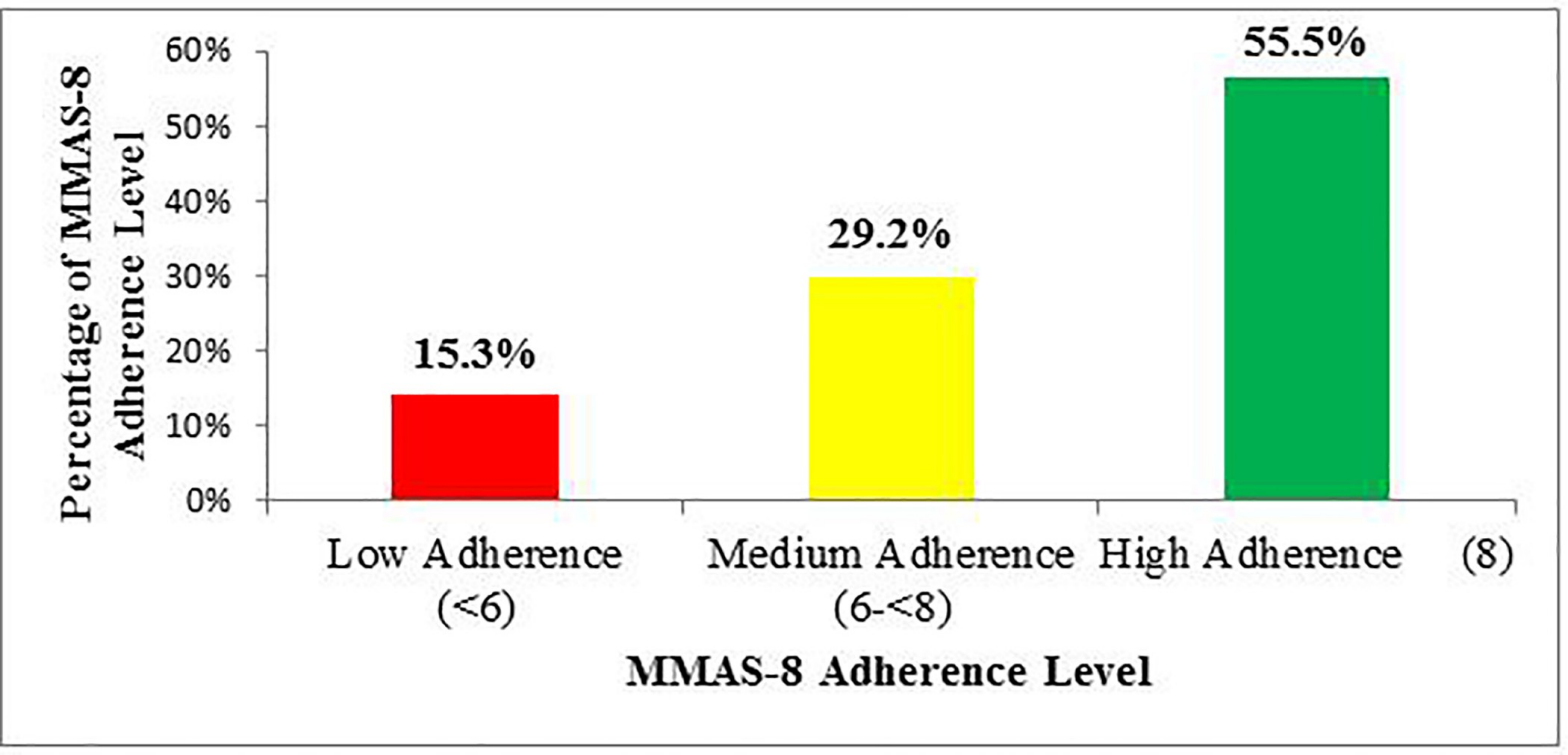
Adherence rate.

Assessment of patients’ responses to the MMAS-8 among those who completed a 3-months follow up showed that 55.5%, 29.2% and 15.3% patients exhibited high, medium and low adherence to Imatinib treatment, respectively, as depicted in Fig 2.

**Fig 3:**
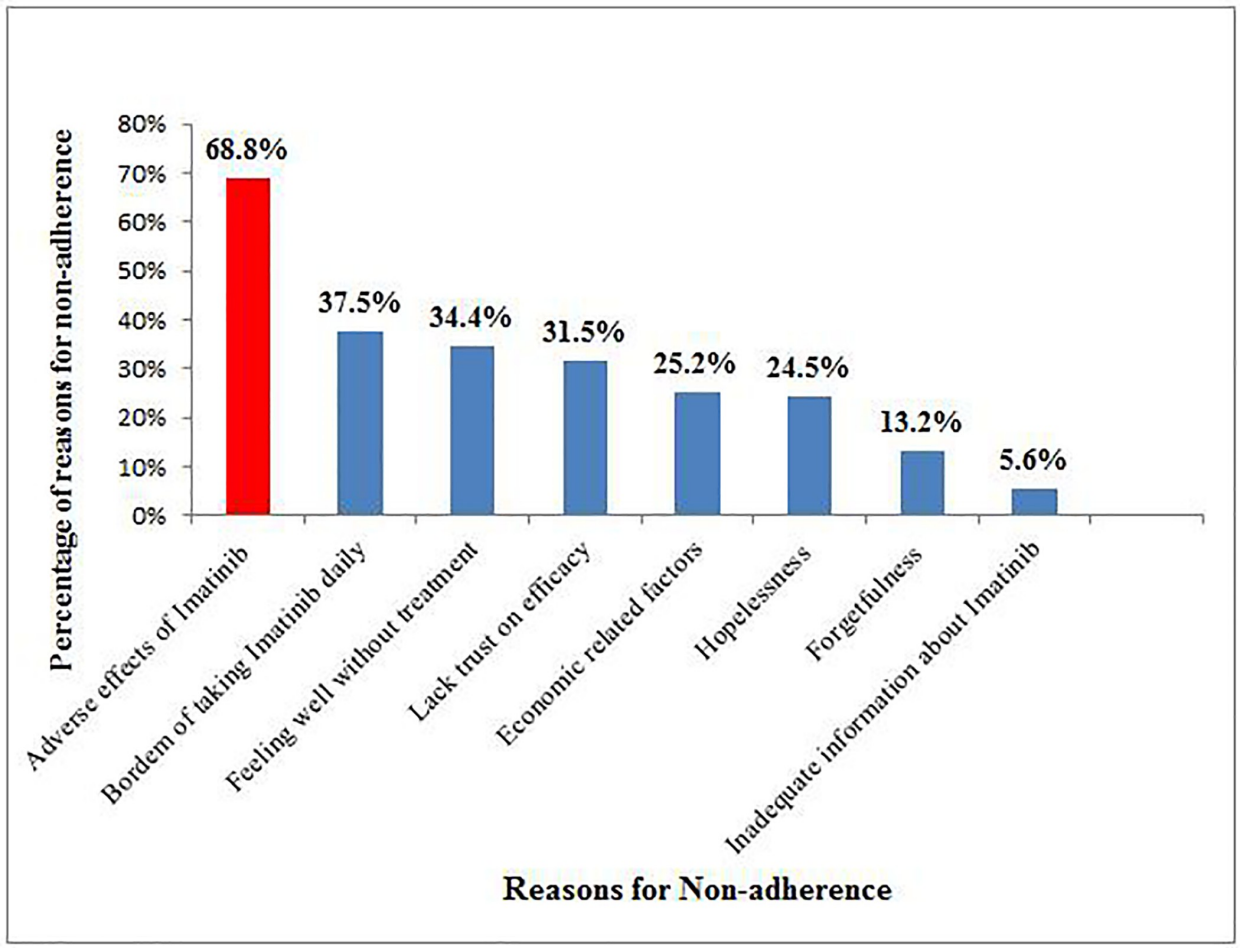
Reasons for non-adherence.

Up on evaluation of the reasons for CML patients’ non-adherence to Imatinib treatment, it was identified that adverse drug events of Imatinib was the main reason for their non-adherence (68.8%). Furthermore, boredom of taking Imatinib daily, feeling well without treatment and lack of trust on efficacy of treatment due to religious beliefs accounted for 37.5%, 34.4% and 31.5% of medication non-adherence, respectively. Forgetfulness and inadequate information about the drug were, however, the least common reasons for non-adherence (Fig 3).

Treatment response at each time line was also assessed based on appointment period given which was at 2 weeks, 1, 2 and 3-months. The median for total number of clinic visits during study period was 4-times (Ranged: 2–8 times). Among 127 patients visited hematology clinic after 2-weeks of Imatinib treatment, 29 (22.8%) of them achieved CHR, of which patients who had WBC count <50 x 10^3^ cells/ mm^3^ at time of initiation of Imatinib accounting for the highest proportion (27, 93.1%). WBC count after 2-weeks of Imatinib treatment was also increased from baseline in 14 (11.1%) of the patients. Majority of the patients who came after 1-month (n = 121) and 2-months (n = 118) of treatment achieved CHR; (73, 60.3%) and (93, 78.8%), respectively. From the 147 newly diagnosed patients, 3 (2%) of them were lost to follow up with unknown status. Overall, among the 144 study participants who completed follow up study, 132 (91.7%) of them achieved CHR at the end of 3-month. Majority of patients (7, 58.3%) who failed to achieve CHR at the end of follow-up were in the WBC count at initiation of Imatinib <50 x 10^3^ cells/ mm^3^ category. In the subgroup analysis most of patients (60, 74.1%) in the <50 x 10^3^ cells/ mm^3^ category were from country side (out of Addis Ababa). The median time for CHR attainment was 6 weeks (Ranged: 2-16weeks) (Table 3).

**Table 3:** Response status to Imatinib treatment among newly diagnosed patients’ with chronic myeloid leukemia at Tikur Anbessa Specialized Hospital from October 1, 2016- November 30, 2017.

Duration of Imatinib Treatment		Response Status based on baseline WBC category*, n (%)					
		≤50k	51-100k	101-150k	≥151k	Total	*p*-value
**2 weeks**^#^	CHR	27(93.1)	0	2(6.9)	0	29(22.8)	
	No CHR	34(40.5)	23(27.4)	14(16.6)	13(15.5)	84(66.1)	
	WBC increased from baseline**	5(35.7)	6(42.9)	0	3(21.4)	14(11.1)	
	Total	66(52.0)	29(22.8)	16(12.5)	16(12.5)	127	*<0*.*0001*^a^
**1 month**^#^	CHR	50(68.5)	11(15.1)	7(9.6)	5(6.8)	73(60.3)	
	No CHR	18(37.5)	15(31.3)	5(10.4)	10(20.8)	48(39.7)	
	Total	68(56.2)	26(21.5)	12(9.9)	15(12.4)	121	*0*.*004*
**2 months**	CHR	52(55.9)	22(23.7)	9(9.7)	10(10.7)	93(78.8)	
	No CHR	10(40.0)	5(20.0)	5(20.0)	5(20.0)	25(21.2)	
	Total	62(52.5)	27(22.9)	14(11.9)	15(12.7)	118	*0*.*245*
**3 months**	CHR	74(56.1)	28(21.2)	14(10.6)	16(12.1)	132(91.7)	
	No CHR	7(58.3)	2(16.7)	3(25.0)	0	12(8.3)	
	Total	81(56.3)	30(20.8)	17(11.8)	16(11.1)	144	*0*.*320* ^a^

*White blood cell count x 10^3^ cells/ mm^3^ found at time of initiation of Imatinib treatment.

**White blood count increased as compared to the preceding complete count test findings and also is part of no CHR category.

^#^ Significant at *p*<0.05

^a^ If fisher exact test is employed, otherwise χ2 test is used.

Based on the results of univariate binary logistic regression analysis, variables such as gender, age, occupation, residence, distance between patients home and institution, educational status, family average monthly income, presence of adverse drug events of Imatinib and comorbidities were included in the multivariable logistic regression analysis. After controlling different demographic and other factors through the use of multivariable logistic regression analysis, this study showed that only adverse drug events, residence, family monthly income, comorbidity and occupational status had significant association with Imatinib treatment adherence. Accordingly, patients who had no experience of any adverse drug events of Imatinib were about six (AOR = 6.12, 95% CI: 2.31, 14.3) times more likely to adhere as compared to those who encountered at least one grade I-IV adverse drug events of Imatinib other than physician led reasons for temporary cessation of treatment. Patients who had an average family monthly income (AOR = 3.43, 95% CI: 1.25–7.74) and high income (AOR = 3.62, 95% CI: 1.09–6.61) were more likely to adhere as compared to those who had very low income.

The study also found that being merchant and employed had a significant association with their level of adherence, where they were about six (AOR = 6.55, 95% CI: 1.98, 44.10) and four (AOR = 4.61, 95% CI: 1.31, 23.86) times more likely to adhere, respectively, as compared with patients who were house wife. Patients who had no comorbid illness were also 4.39 times more likely to adhere to their treatment when compared to those who had at least one comorbid illness (AOR = 4.39, 95% CI: 1.16, 16.64). On the other hand, patients who came from country side (AOR = 0.29, 95% CI: 0.02, 0.84) were less likely to adhere compared to those from Addis Ababa (Table 4).

**Table 4:** Univariate and multivariable binary logistic regression analysis of predictors of Imatinib treatment non-adherence among Ethiopian patients with CML.

Variables		Adherence		COR, 95%CI	AOR, 95%CI
		Suboptimal Adherence	Adherent		
Gender					
	Female	31(48.4)	28(35.0)	1.00	1.00
	Male	33(51.6)	52(65.0)	1.63(0.83, 3.19)	1.02(0.71, 2.77)
Occupation					
	House Wife	15(23.4)	8(10.0)	1.00	1.00
	Farmer	13(20.3)	12(15.0)	1.44(0.46, 4.52)	1.63(0.37, 7.12)
	Merchant/Trader	9(14.1)	17(21.3)	2.94(1.15, 9.42)*	6.55(1.98, 44.10)*
	Employee	12(18.8)	25(31.3)	3.24(1.24, 9.60)*	4.61(1.31, 23.86)*
	Retired	8(12.5)	7(8.8)	1.12(0.365, 5.07)	1.65(0.48, 5.38)
	Students	7(10.9)	11(13.8)	1.71(0.52, 5.67)	2.76(0.56, 13.5)
Educational Status					
	Can’t read and write	22(34.4)	20(25.0)	1.00	1.00
	Primary Education	18(28.1)	19(23.8)	1.22(0.51, 2.90)	1.41(0.2, 1.92)
	Secondary Education	9(14.1)	13(16.2)	1.58(0.56, 4.46)	1.6(0.84, 2.54)
	Higher Education	15(23.4)	28(35.0)	1.85(0.79, 4.35)	1.42(0.59, 1.86)
Family Income					
	Low (<1500)	42(65.6)	26(32.5)	1.00	1.00
	Average (1501–5000)	20(31.3)	40(50)	2.92(1.68, 4.82)*	3.43(1.25, 7.74)*
	High (>5001)	2(3.1)	14(17.5)	3.01(1.25, 5.39)*	3.62(1.09, 6.61)*
Place of residence					
	Addis Ababa	8(12.5)	29(36.3)	1.00	1.00
	Out of Addis Ababa	56(87.5)	51(63.7)	0.42(0.19, 0.91)*	0.29(0.02, 0.84)*
Presence of Comorbidity					
	At least One	9(14.1)	3(3.7)	1.00	1.00
	None	55(85.9)	77(96.3)	2.72(1.21, 8.80)*	4.39(1.16, 16.64)*
Adverse effects					
	At least one	56(87.5)	54(67.5)	1.00	1.00
	None	8(12.5)	26(32.5)	4.34(1.35, 7.78)*	6.12(2.31, 14.3)*

COR = Crude odd ratio, AOR = Adjusted odd ratio

*Statistically significant at *P* < 0.05

## Discussion

This is the first prospective cohort study conducted to gain knowledge about adherence and determinates of poor adherence in newly diagnosed patients with CML in Ethiopia. The findings of this study could be used in treatment decisions, to design innervations to improve Imatinib treatment adherence and will also be used as baseline data source for other studies.

Imatinib was given for free for all the study participants. Moreover, despite its tolerability profile compared to other anti-cancer drugs, the high efficacy and the convenience of oral administration; which are convincing reasons for patients with CML to regularly take their medication, still adherence was a significant problem in our patients.

In keeping with the general perception of physicians about patient adherence to Imatinib treatment as a major issue; the adherence rate in our study participants was 55.6%. This finding is comparable with previous studies conducted in India (45%) [12] and Qatar (61%) [13]. On the contrary, adherence rate was considerably lower in our study as compared to a Swedish (97%) [14] and a meta-analysis study (75.2%) [15]. The low adherence rate in this study is assumed to be the fact that we have a single treatment center for the whole country with a population of more than 100 million, differences in literacy level, and also that most of our study participants were from the country side. The additional reason for non-adherence may be the frequent experience of adverse drug events mainly GI-related that was documented in almost 69% of the study participants (Fig 2). Hence, more effort needs to be exerted to increase treatment adherence of CML patients, so that they could benefit and achieve treatment milestones.

Our study showed that, the odds of being adherent in patients who didn’t experienced any adverse drug events were about 6-times more likely to adhere to their medication than those who experienced at least one adverse drug event. This finding is in line with a qualitative study conducted in Taiwan [16] and India [17] that demonstrated Imatinib-related adverse drug events were the most common hindering reason for adherence. Numerous studies also support the findings of our study that adverse drug events had a significant inverse association with Imatinib treatment adherence [18–20]. Moreover, patients who lived near to the treatment center were more likely to adhere compared to those who were from countryside. Although Imatinib is given free of charge for all patients, the possible explanation for this difference may be that patients from the countryside have to travel long distances with extra transportation costs and other expenses related to meals and hotel.

On the other hand, as the family income increased, patients were found to be more likely to adhere to their medication. This is supported by other studies which reported that economic status had a significant association with Imatinib adherence [12,20]. Interestingly, housewives were less likely to adhere to treatment in our study. Few of the reasons may be that the housewives are economically dependent on their husbands and therefore may not routinely come to the treatment center, and the adverse effects might be more pronounced in females compared to males due to physiologic differences.

The present study also revealed that presence of comorbid conditions were significantly predictive of non-adherence that is in keeping with other studies [9,17,20]. This could be due to pill burden prescribed for the other comorbid conditions. This is clearly illustrated by the 5 patients who had HIV in our study.

In this study, patients with poor adherence reported several reasons for not taking their medication. Similar to other studies [12,20,21]; the most common reasons were found to be adverse drug events of Imatinib, boredom of taking Imatinib daily, feeling well without treatment and perceived lack of trust on the effectiveness of treatment. Similarly, the above mentioned two qualitative studies revealed that adverse drug events were identified as the main obstacle for medication adherence [16,22]. Forgetfulness and inadequate information about the drug were, however, the least common reasons for non-adherence.

Adherence was also found to be a major determinant of CML treatment success. Study participants who had Morisky high adherent and medium adherent scores were about nine (AOR = 8.6, 95% CI: 4.32–11.1) and seven (AOR = 6.9, 95% CI: 3.1–10.7) times, respectively, more likely to achieve CHR than those who were low adherent.

In conclusion, poor adherence to treatment should be considered as a possible reason for patient non-or reduced response to Imatinib before considering these patients to be Imatinib-resistant and switching them to next-line treatment. Hence, identifying specific barriers for each patient and adopting a suitable technique to overcome the obstacles of adherence will be necessary to improve treatment outcome. Health care providers have a significant role in the improvement of medication adherence in their daily practice.

As a limitation, since self-report was used for the assessment of adherence, the present study was dependent on the assumption that patients who claimed to adhere to Imatinib treatment have actually adhered. Finally; the sample size of our study was small because of time constraint and may have affected the power of the study.

## Supporting information

S1 File Data collection tool. (PDF)
