# Supplementary material for: Prevalence and determinants of non-adherence to Imatinib in the first 3-months treatment among newly diagnosed Ethiopian’s with chronic myeloid leukemia
Source: PLoS One. 2019 Mar 7;14(3):e0213557. doi: 10.1371/journal.pone.0213557 (PMC6405163; doi:10.1371/journal.pone.0213557)
Supplement: S1 File — (PDF) [file pone.0213557.s001.pdf]

Data Collection Tool

Annex I: Structured Questionnaire

Part I: English Version of Information Sheet

Dear participant, Good Morning/Afternoon

Introduction

My name is ________________. I am a member of the study that is carried out at Tikur Anbessa Specialized Hospital, Ambulatory Hematology Clinic; Addis Ababa, Ethiopia entitled "Prevalence and Determinants of Non-adherence to Imatinib in the First 3-Months among Newly Diagnosed Ethiopian Patients with Chronic Myeloid Leukemia".

Objective

The main purpose of this study is to assess "Prevalence and Determinants of Non-adherence to Imatinib in the First 3-Months among Newly Diagnosed Ethiopian Patients with Chronic Myeloid Leukemia". Your input will be extremely valuable as the information will be used to assess the medication use and adherence to identify gaps in treatment outcome.

Expected Outcomes and/or Benefits

At the end of the study, the adherence and factors associated with poor adherence will be evaluated. Therefore, the study will identify and investigate the main gaps and challenges associated with adherence and will propose the feasible recommendations and may benefit you directly or indirectly by improving the CML treatment in the hospital.

Thank you so much in advance.

Part II: English Version of Informed Consent Form

Everything from your information and records would be completely confidential to the research and the data are stored without your name and only used for the purpose of this study. None of this would affect the care you receive from Hematology Ambulatory clinic of TASH, but will help in future planning for the hospital. No identifying names or characteristics will go into my report, so you may share your thoughts openly. Additionally, taking part in this study is completely voluntary. It is your choice whether to participate or not. You may skip any questions that you do not want to answer. Please ask me to stop as we go through the information and I will take time to explain. I would be grateful if you could sign the attached form to say you have no objections to our accessing any records and interviewing you. Would you be willing to assist me by having a 10-15 minutes'?

If the interviewee responds "Yes", Please proceed and let him/her to sign or replies "No" gratitude him/her and quit the interview. If you have any questions concerning the study, please call Atalay Mulu (+251) 23295462.

____________________ ______________________

Signature of respondent **Signature of interviewer**

Date: ______________________ (Day/month/year)

Principal Investigator: Atalay Mulu

Addis Ababa University, CHS, School of Pharmacy, Department of

Pharmacology and Clinical Pharmacy

Email: Atalay.mulu@aau.edu.et

Part III: Data Collection form from Patient Interview

**Socio-Demographic Characteristics**
**Age:_____________________(in years)**
**Sex:**<br>❑ Male ❑ Female
**Marital Status**<br>❑ Single ❑ Married ❑ Divorced ❑ Widowed/er
**Educational Level**<br>❑ Can't read & write ❑ Can read and write without formal education<br>❑ Primary Education(1-8) ❑ Secondary Education(9-12)<br>❑ Diploma and Above ❑ Others
**Place of Residence**<br>❑ Addis Ababa ❑ Out of Addis Ababa
**Region: ______________________(Write in words where they are from)**
**Occupation**<br>❑ Unemployed ❑ House wife ❑ Student<br>❑ Farmer ❑ Daily laborer ❑ Employed<br>❑ Merchant ❑ Retired ❑ Other(s) [Specify]
**Monthly Income (ETB): _____________** (Write in number)<br>❑ Very Low (≤ 860 Birr) ❑ Above average (3001-5000 Birr)<br>❑ Low (861-1500 Birr) ❑ High (≥5001 Birr)<br>❑ Average (1501-3000Birr)

Part IV: Adherence Assessment Tool

Morisky Medication Adherence scale (8-item) was employed to measure adherence status.

Part V: Reasons for Non-Adherence

If the finding for the MMAS-8 score is <8 (or any suspicion of things which affects adherence to treatment), what do you think are the factors?

❑ Unintentional (forgetfulness, carelessness)

❑ Intentional, due to lack of trust on the treatment (personnel beliefs about taking medication such as cultural beliefs)

❑ Costs other than medication price (transport cost, cost for home and bed accommodation while coming for medication refill)

❑ Hopelessness (consider taking medication lifelong while it is not cured)

❑ Lack of support from family or friend

❑ Lack of support from health professionals

❑ Distance from AA or hospital

❑ Others, specify____

Part VI: Safety of Imatinib

What are the adverse effects you faced due to your medication or review on patient chart?

❑Edema/Fluid retention ❑ Headache ❑ Nausea/Vomiting

❑Fatigue ❑ Muscle pain ❑ Epigastric pain/Burning sensation

❑Rash ❑ Diarrhea ❑ Skin rash

❑ Facial Puffiness ❑ Skin color change ❑ Others____________________

Based on answers provided in above, do the above adverse events affect your medication taking behavior? ❑ Yes ❑ No

If yes in question 3.4; how often did you miss your dose?

❑Never ❑Rarely ❑ Sometimes ❑ Often

Annex II: Data Abstraction format from Patient Chart

Part I: Presence of Co-morbid Illnesses

1. Are there any Co-morbid conditions other than CML? ❑ Yes ❑ No
2. If yes for question number 1, what type of co-morbid condition/s is this/are they? ____
3. Is there any medications taken by the patient? Please list all the medications ____

I. Treatment History

1. Did the Patient take hydroxyurea? ❑ Yes ❑No
2. If yes for Qn no 1; dose, frequency and duration of treatment:- ____
3. Difference between confirmed diagnosis of CML and initiation of Imatinib, in weeks: ____
4. **Daily dose of Imatinib:**
   ❑ 300mg ❑ 400mg ❑ 600mg ❑ 800mg
5. **About Regimen/dose change History**

I. Was the dose/ regimen changed? ❑ **Yes** ❑ **No**

II. **If yes to Qn No I,**

When (after initiation of Imatinib in weeks)________________________

Why?

a. Dose decreased, because the patient responds to the initial dose and changed to maintenance treatment.

b. Dose decreased, because the patient didn't tolerate

c. Dosed increased, because the patient didn't respond to current treatment

d. Drug discontinued because patient fulfills for Imatinib discontinuation.

6. **If the answer is "d", Reasons for temporary treatment discontinuation**

____

7. **For how long, treatment discontinued (In weeks)________________________**

8. **Supportive managements given for the patient**

   1. ______________________________
   2. ______________________________
   3. ______________________________

9. **Follow up findings**

**CBC**	At 2wks	At 1month	At 2-month	At 3-month
WBC x 10^3 cells/ mm^3				
Neutrophil, %				
ANC				
Lymphocyte, %				
Basophils, %				
Platelet count x 10^3 cells/ mm^3				
RBC x 10^6 cells/ mm^3				
HCT, %				
Hgb, g/dl				
**Clinical Findings**				

Splenomegaly (Yes/No)				
Hepatomegaly (Yes/No)				
CHR status(Yes/No)				
Are/is there other clinical findings? If yes Specify				

Annex III: የአማርኛ መጠይቅ ቅፅ

ቅጽ 1: የጥናቱ መረጃ ቅጽ

ቀን፦___________________

ውድ የቃለ መጠይቅ ተሳታፊ፤ እንደምን አደሩ/ዋሉ? የጥናቱ መግቢያ ስሜ ________________________________ ይባላል፡፡ በጥቁር አንበሳ ስፔሻላይዝድ ሆስፒታል ሄማቶሎጅ ክፍል ዉስጥ በተመላላሽነት እንደ አዲስ Imatinib (Gleevec) የተባለዉን መድኃኒት ለጀመሩ የCML ህሙማን ስለ መድሀኒት አወሳሰድ እና የህክምና ዉጤት ሁኔታ የ 3-ወር የክትትል የድህረ ምረቃ ጥናት ” ሲሆን የጥናቱም አባልም ነኝ፡፡ በተለይ የእርስዎ የመድኃኒት አጠቃቀም በጥናቱ ላይ በደንብ ከሚጠኑ ነጥቦች አንዱ ነዉ፡፡

የጥናቱ አላማ፦

የዚህ ጥናት ዋና አላማው Imatinib(Gleevec) የህክምናዉ ዉጤት ምን እንደሚመስል ፡ መድኃኒት እንዴት እንደሚጠቀሙት፣ በታዘዘው መሰረት በአግባቡ እንዴት መድኃኒትዎን እንደሚወሰዱት እና መድኃኒትዎን ሁል ጊዜ እንዳይወሰዱ የሚያደርጉ ዋና ዋና ክፍተቶችን በመለየት፤ የመፍትሄ ሀሳቦችን ማቅረብ ነው፡፡

ከጥናቱ የሚጠበቁ ውጤቶች/ጥቅሞች

በዚህ ጥናት ላይ የእርስዎ የሚጥል መድኃኒቶች በታዘዘው መሰረት በአግባቡ የአወሳሰድና የአጠቃቀም ክህሎት በድንብ ይጠናሉ፡፡ መድኃኒትዎን ሁል ጊዜ እንዳይወሰዱ የሚያደርጉ ዋና ዋና ክፍተቶችን በመለየት፤ የመፍትሄ ሀሳቦችን ማቅረብ ነው፡፡በተጨማሪም ከጥናቱ በሚገኙ ግኝቶች የሚጥል ህክምና ዉጤትን በተወሰነ መልኩ ለማሻሻል እንደሚቻል በመገመት፤ እርስዎ የጥቅሙ ተቋዳሽ ይሆናሉ ብለን እናምናለን፡፡ ስለዚህ የእርስዎ ቅንና ሓቀኛ መረጃ ለጥናቱ እጅግ በጣም ወሳኝ ነው፡፡

የተከበረ ጊዜዎ ስለሰጡን እጅግ በጣም እናመሰግንዎታለን፡፡

ቅጽ 2፡ በቃለ መጠይቅ ለመሳተፍ የፈቃደኝነት ቃል መቀቢያ ቅጽ

በCML ታካሚዎች ዉጤት እንዲሁም ተያያዥ ጉዳዮች ላይ ያጠነጠነ እና ከመድሃኒቱ አወሳሰድ እንዲሁም ከህክምናዉ ጋር ተያይዘዉ ያሉ ችግሮችን በመቅረፍ የበሽታዉን የህክምና ዉጤት ለማሻሻል አላማ አድርጎ የተነሳ ጥናት ነው፡፡ መሰብሰቢያ ቅፁም ይህንኑ አላማ አድርጎ የተዘጋጀ ነዉ፡፡ በመሆኑም ከእርስዎ፡ ከእርስዎ ካርድ ላይ እና ከሀኪምዎ መረጃ ለመውሰድ እንፈልጋለን፡፡በዚህ ጥናት ለመሳተፍም ሆነ ላለመሳተፍ መወሰንዎ በሆስፒታሉ ዉስጥ ለሚያገኙት ማንኛዉም አገልግሎት ላይ ምንም አይነት ተጽዕኖ የማይኖረዉ ሲሆን ተሳትፎዉንም በማንኛዉም ሰዓት ማቋረጥ ወይም ጥያቄዎችን አለመመለስ ይችላሉ፡፡ በዚህ ጥናት የእርስዎ መረጃ ሙሉ በሙሉ በምስጥር የተጠበቀና ለምርምሩ አላማ ብቻ የሚዉል ነው፡፡ በተጨማሪም የእርስዎ ተሳታፊነት በፈቃደኝነት የተመሠረተ ነው፡፡ የጥናቱ አላምውን ተረድተውና ጊዜዎን ሰውተው፤ ከ 10-15 ደቂቃዎች ለሚፈጅ ቃለ-መጠይቅ እውተኛው መረጃ ለመስጠት ፍቃደኛ በመሆንዎ በቅድሚያ አመሰግናለሁ፡፡ አዎ ከሆነ፣ ያስፈርሙ እና ይቀጥሉ፤ ካልሆነ ወደሚቀጥለዉ ተጠያቂ ይሂዱ፡፡

____________________________ ____________________________

የቃለ መጠይቅ የቀረበለት ሰው ፊርማ የቃለ መጠይቅ አቅራቢ ፊርማ

በየትኛው ጊዜ ጥያቄ ካለዎት አታላይ ሙሉ ብለው በ ስ.ቁ. (+251) -92295462 ወይም በ ኢ-ሜይል atalay.mulu@aau.edu.et ይጠይቁን፡፡

የተከበረ ጊዜዎ ስለሰጡን እጅግ በጣም እናመሰግንዎታለን፡፡

ዋና አጥኚ:

አዲስ አበባ ዩኒቨርሲቲ፣ ጤና ሳይንስ ኮሌጅ፤ ፋርማሲ ት/ቤት፤ ፋርማኮሎጅና ክሊኒካል ፋርማሲ ትምህርት ክፍል

ኢ-ሜይል: atalay.mulu@aau.edu.et

ስ/ቁ፡ 09 23 29 54 62

Socio-Demographic Characteristics
**እድሜ:_________________________(በዓመት)**
ፆታ:<br>❑ወንድ ❑ሴት
**የጋብቻ ሁኔታ**<br>❑ያላገባ/ች ❑ያገባ/ች ❑አግብታ/ቶ የፈታ/ች ❑ ባል/ሚስት የሞተባት/ችበት
**የትምህርት ደረጃ**<br>❑ ማንበብም ሆነ/መፃፍ የማይችል ❑ ማንበብም እና መፃፍ የሚችል ያለ ዘመናዊ ትምህርት<br>❑ የመጀመሪያ ደረጃ (1-8) ❑ ሁለተኛ ደረጃ (9-12)<br>❑ ድህረ ምረቃ የጨረሰ/ች ❑ ሌሎች ___________________________
**የሚኖሩበት ቦታ**<br>❑ አዲስ አበባ ❑ ከአዲስ አበባ ዉጭ
**የመጡበት ክልል: __________________________(በፅሁፍ ይፃፍ)**
**ስራ**<br>❑ ስራ አጥ ❑ የቤት እመቤት ❑ ተማሪ<br>❑ አርሶ/አርብቶ አደር ❑ የቀን ሰራተኛ ❑ ተቀጣሪ(ወርኃዊ ተከፋይ)<br>❑ ነጋዴ ❑ ጡረተኛ ❑ ሌሎች [ይገለፀ] ___________
**ወርሀዊ የቤተሰብ ገቢ (በብር): ________________** (በቁጥር ይጻፍ)<br>❑ በጣም ዝቅተኛ (≤ 860 ብር) ❑ ከአማካይ በላይ (3001-5000 ብር)<br>❑ ዝቅተኛ (861-1500 ብር) ❑ ከፍተኛ (≥5001 ብር)<br>❑ አማካይ (1501-3000ብር)

Part 1: <u>ሞሪስኪ" መድኃኒትን በታዘዘው መሰረት በአግባቡ ስለመውሰድ" መለኪያ- 8</u>

እርስዎ እነደነገሩን/ እነደሚታወቀዉ ለCML ማከሚያ የሚሆን Imatinib የሚባል መድሀኒት እየወሰዱ ይገኛሉ፡፡ ነገር ግን ይህንን ወይም ሌላ መድሀኒት የሚወስዱ ብዙ ሰዎች ከሚያጋጥማቸዉ እና ካላቸዉ ልምድ ሥለ መድሃኒት አወሳሰዳቸዉ ባህሪ ብዙ ነገር ስለሚሉ እኛ ደግሞ የእረስዎን ማለትም የImatinib መድሀኒት አወሳሰድ ባህሪ ለማወቅ እንፈልጋለን፡፡ ስለዚህ እባክዎትን እርስዎ የሚሰማዎትን ትክክለኛ ነገር በሚቀጥሉት መጠይቆች መሰረት ይንገሩን፡፡

	ሞሪስኪ" መድኃኒትን በታዘዘው መሰረት በአግባቡ ስለመውሰድ" መለኪያ- 8<br>ጥያቄዎች	አዎ (0)	አይደለም (1)
1	አንዳንድ ጊዜ መድኃኒትዎን ረስተው ሳይወሰዱ ቀርተው ያውቃሉ?		
2	ሰዎች አንዳንድ ጊዜ ከመርሳት ውጪ ባሉት የተለያዩ ምክንያቶች መድኃኒታቸውን ሳይወስዱ ይቀራሉ፡፡ ባለፉት ሁለት ሳምንታት፣ መድኃኒትዎን ሳይወስዱ የቀሩበት ቀናቶች ነበሩ'?		
3	ሐኪምዎን ሳይነግሩ፣ መድኃኒትዎን እየወሰዱ ህመምዎ ሲባባስ፣ መድኃኒትዎን አቋርጠው ያውቃሉ?		
4	በጉዞ ምክንያት ወይም ከቤትዎ አርቀው ሲጓዙ፣ አንዳንድ ጊዜ መድኃኒትዎን (ወደጉዞው) ረስተውት ሳይወስዱት ያውቃሉ?		
5	በትላንትናው ዕለት ሁሉንም መድኃኒትዎን ወስደውታል?		
6	ህመምዎ ሲሻልዎት (የህመምዎ ስሜቶች ሲጠፉ) አንዳንድ ጊዜ መድኃኒትዎን አቋርጠው ያውቃሉ?		
7	በየቀኑ መድኃኒት መውሰድ፣ ለአንድ አንድሰዎች ምቾት አይሰጣቸውም፡፡ እርስዎ በየቀኑ፣ እንድሁም አንድም ሰዓት ሳያዛንፉ መድኃኒትዎን መውሰድዎ፣ የመሰላቸት ስሜት ተሰምቶት ያውቃል?		

8	መድኃኒትዎን አስታውሰው ለመውሰድ ምንያክል ይቸገራሉ?<br>❑ ጭራሽ ተቸግሬ አላዉቅም(1)<br>❑ ዕለታት አንድ ጊዜ እቸገራለሁ (0.75)<br>❑ አልፎ አልፎ እቸገራለሁ (0.5)<br>❑ አብዛኛው ጊዜ እቸገራለሁ (0.25)<br>❑ ሁል ጊዜ እቸገራለሁ (0)<br>❑**Low (<6)** ❑ **Medium (6-<8)** ❑**High(8)**		

Part 2: **መድሀኒትን በታዘዙት መሠረት/በትክክለኛዉ መንገድ እንዳይወስዱ ያደርጋሉ ተብለዉ የታሰቡ ችግሮችን ማወቂያ**

2.1. በሞርስኪ-8 መጠይቅ መልስ መሰረት Adherence level ከ8-በታች ላሉት ታካሚዎች የሚጠየቅ ጥያቄ፡ ከሚከተሉት የመድሃኒት አወሳሰድን ያዉካሉ ተብለዉ ከተቀመጡ ምርጫዎች መካከል እርስዎን በታዘዙት መሰረት እንዳይወስዱ ያስቸገርዎት የትኞቹ ናቸዉ (ከአንድ በላይ መልስ ይቻላል)፡፡

በመርሳት	❑ ተስፋ በመቁረጥ
❑ በቸልተኝነት/በግዴለሽነት	❑ ሁሌ መድሀኒት መዉሰድ ስለሚሰለቸኝ
❑ባህላዊ እና ኃይማኖታዊ በሆኑ ምክንያቶች ተጨማሪ ህክምና በመፈለግ፡ ህክምናዉን ሙሉ በሙሉ ስለማላምንበት	❑ ህክምና ቦታ ያሉት የጤና ባለሙያዎች ድጋፍ ስለማያደርጉሊኝ
❑ ቤተሰቦቼ/ጓደኞቼ በተለያዩ ነገሮች ድጋፍ ስለማያደርጉሊኝ	❑ በመድኃኒቱ የጎንዮሽ ምክንያት
❑ የምመጣበት አካባቢ ሩቅ ስለሆነ በቀጠሮየ ቀን ለመምጣት ገንዘብ ስለሚቸግረኝ	❑ ሌሎች ካሉ በፅሁፍ ይገለፅ ______________________________ ____

2.2. Imatinib የተባለዉን መድሀኒት ሲወሰድ ያጋጥማሉ የተባሉ የጎንዮሽ ጉዳቶችን ማወቂያቅፅ፡

2.2.1. እባክዎትን መድሀኒቱን ሲወስዱ ወይም መዉሰድ ከጀመሩ በኂላ ያጋጠምዎትን ችግሮች ይንገሩን(ከአንድ መልስ በላይ መልስ መመለስ ይቻላል)፡፡

- ❑ የዉነትዎት እብጠት
- ❑ የማቅለሽለሽ ስሜት/ ማስታወክ
- ❑ ድካም
- ❑ ተቅማጥ
- ❑ ሰዉነትን ማሳከክ?
- ❑ የ ጨ ጓ ራ / የ ሆ ድ ህ መ ም
- ❑ ራስ ምታት
- ❑ ሰዉነት ማሳከክ
- ❑ የመገጣጠሚያ ህመም
- ❑ የቆዳ ቀለም መቀየር
- ❑ የፊት ቆዳ ማፈግፈግ
- ❑ ሌሎች______________________

2.2.2. ከላይ በሰጡን መልስ መሰረት፡ የዘረዘሯቸዉ የጎንዮሽ ጉዳቶች የመድሀኒት አወሳሰድዎትን አዉከዎት/በታዘዙት ትክክለኛ አወሳሰድ እንዳይወስዱ አድርጎዎት ነበር?

❑ አዎ ❑ የለም

2.2.3.ከላይ በ2.2.2. የሰጡት መልስ አዎ ከሆነ፡ ምን ያህል ጊዜ መድሀኒትዎትን ሣይወስዱ ቀርተዋል/ዘለዋል

- ❑ ምንም አልዘለልኩም
- ❑ አንዳንድ ጊዜ
- ❑ከዕለታት አንድ ጊዜ
- ❑ አብዛኛው ጊዜሁል ጊዜ
